# Effects of Extensor Digitorum Longus and Tibialis Anterior Taping on Balance and Gait Performance in Patients Post Stroke

**DOI:** 10.3390/healthcare10091692

**Published:** 2022-09-05

**Authors:** Kyun-Hee Cho, Shin-Jun Park

**Affiliations:** 1 Department of Physical Therapy, Gangdong University, Eumseong-gun 27600, Korea; 2 Department of Physical Therapy, Suwon Women’s University, Hwaseong-si 18333, Korea

**Keywords:** ankle joint, taping, stroke, gait, balance

## Abstract

The purpose of this study was to investigate the effects of extensor digitorum longus taping (EDLT) and tibialis anterior taping (TAT) on balance and gait performance in patients post-stroke. The study included 40 stroke patients randomly assigned to two intervention groups: the EDLT group and the TAT group. Therapeutic taping was applied to the extensor digitorum in the EDLT group and applied to the tibialis anterior in the TAT group. Balance variables were measured using BioRescue equipment, and gait variables were measured using G-walk equipment. Balance and gait variables were significantly increased in both the EDLT and TAT groups after the intervention, but there were no significant differences between the two groups. Therefore, we concluded that eversion (EDLT) or inversion (TAT) through taping did not affect the outcome. Only dorsiflexion affects gait speed increase post-stroke. As a result of this study, extensor digitorum longus taping and tibialis anterior taping were taping methods with no difference in the improvement of balance ability and gait performance.

## 1. Introduction

Restoring balance and gait ability through ankle control is one of the indicators for reducing further damage and disability in patients post-stroke [1]. In the clinical field, ankle control recovery is essential for rehabilitation post-stroke [2]. 

Patients post-stroke frequently experience ankle control disorders [3,4,5,6,7]. Ankle control disorders cause weakness [5], spasticity and contracture [6], stiffness [3,4], and sensory dysfunction [7]. Dorsiflexor weakness exacerbates balance disturbances [1] and affects gait speed [5]. Specifically, because the dorsiflexor mainly acts during the initial contact [8] and swing phase during the gait cycle [9], the gait speed is reduced when this muscle is weakened [5].

Representative dorsiflexors include the tibialis anterior and extensor digitorum longus [10]. In addition to their functions in dorsiflexion of the ankle joint, the tibialis anterior acts in ankle dorsiflexion and inversion. The extensor digitorum longus acts in ankle dorsiflexion and eversion as well as extends toes 2–5. [10]. Stroke patients are characterized by a shorter medial gastrocnemius than a lateral gastrocnemius [11,12]. Shortening of the medial gastrocnemius results in plantar flexion and inversion of the ankle joint, which causes equinovarus [13]. Therefore, the activity of the extensor digitorum longus during eversion and dorsiflexion in the swing phase of the gait cycle is crucial [9].

In previous taping studies to improve dorsiflexion in patients post-stroke, Kinesio taping was applied to the extensor digitorum longus and tibialis anterior [14,15,16], or to the tibialis anterior alone [17,18,19]. Taping is an effective intervention method to improve balance and gait ability in stroke patients who have difficulty performing voluntary movements due to movement restrictions such as muscle weakness, spasticity, and contracture [16,19,20,21,22]. Recovery of balance ability through taping is associated with recovery of ankle strategy due to increased dorsiflexion joint range of motion [18]. The tibialis anterior taping promotes the activity of the tibialis anterior muscles when the body moves backward, and taping the calf muscles promotes the activity of the calf muscles when the body moves forward, which helps to maintain balance [19]. AFO orthosis (ankle-foot-orthosis) is used clinically for dorsiflexion fixation. Since the orthosis is fixed rather than functional movement of the ankle, muscle weakness can occur, and it is heavy [23]. On the other hand, taping can compensate for the shortcomings of orthosis because it is light and helps functional movements. However, despite the evidence that such taping is effective, most attempts to improve the balance and walking ability of stroke patients by taping the ankle have focused on attaching tape to the tibialis anterior and extensor digitorum simultaneously or only to the tibialis anterior [14,15,16,17,18,19]. No domestic or foreign studies have individually examined whether the extensor digitorum longus taping (EDLT) treatment or the tibialis anterior taping (TAT) treatment is more effective for balance and gait ability improvement.

Consequently, a more specific muscle-taping method was required to clarify therapeutic effects associated with the taping attachment site. In this study, the effects on balance and gait ability were assessed after individual application of EDLT and TAT treatments. In addition, a taping method for ankle dorsiflexion in patients post-stroke was investigated.

## 2. Materials and Methods

### 2.1. Study Procedure 

This study was a double-blind, randomized controlled trial. Forty subjects were randomly assigned to either the EDLT group (*n* = 20) or the TAT group (*n* = 20). The participants were unaware of the intervention effect and group assignments.

Evaluation was performed by a physical therapist with more than five years of experience who was not aware of the purpose and effectiveness of this study. In the initial evaluation, measurements were made before tape was applied, and in the post-evaluation, and measurements were made 24 h later. All tapings were applied for 24 h and were removed after post-evaluation. The evaluation sequence involved measuring the balance variable and subsequently measuring the gait variable after a 10 min rest to prevent fatigue. A study assistant stood by during the measurement to prevent falls.

The taping intervention in the EDLT and TAT groups was performed in the evening at the end of the treatment routine by a physical therapist with more than 1 years of experience. A treatment routine consisting of neurodevelopmental therapy (NDT), mat, and gait treatment was provided to both groups according to the treatment schedule. This study was approved by the Institutional Review Board of Yongin University (2-1040966-AB-N-01-2101-HSR-245-1).

### 2.2. Sample Size Calculation 

G*Power software (G*Power 3.1, Heinrich Heine-Universität, Düsseldorf, Germany) was used to calculate sample size. Based on a pilot study with 10 subjects, an effect size of 0.45 (calculated as partial η^2^ = 0.167) was obtained for gait speed, which is the primary variable. Using the pilot study effect size of 0.45, we determined that a significance level of 0.05, a power of 0.80, and a total of 32 study subjects were required. Consequently, a total of 40 study subjects were recruited (excluding dropouts) for the study.

### 2.3. Participants 

This study was conducted on patients post-stroke admitted to a hospital in Gyeonggi-do. Subjects were recruited through notices on hospital bulletin boards. All participants voluntarily participated in the study and signed the study consent form. The specific conditions for subject selection were as follows: those who had been diagnosed post-stroke for more than 6 months by a doctor (chronic post-stroke), those with a score of 24 or higher on the Korean mini-mental state examination (K-MMSE), those who could independently walk more than 10 m (without orthosis or cane), subjects with ankle joint modified Ashworth scale (MAS) of grade 1 or 2, subjects with Brunnstrom recovery stage 4 or 5, subjects with no orthopedic surgery of the ankle, and subjects with no medical or surgical history. Patients were excluded if they experienced the following: pain in the ankle, an allergic skin reaction to tape attachment, and cerebellar and vestibular ataxia that may affect the ability of balance.

### 2.4. Intervention

#### Extensor Digitorum Longus and Tibialis Anterior Taping Method

**Figure 1:**
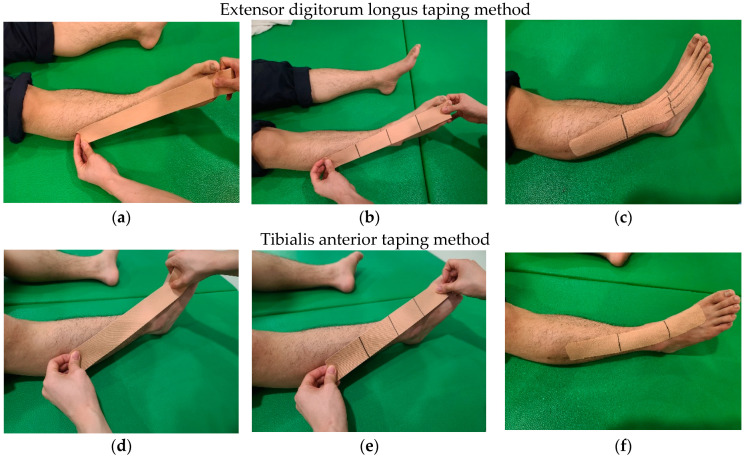
Diagram of taping method is as follows: (**a**) Measure muscle length with tape; (**b**) divide the length of the tape into 4 equal parts; (**c**) attach to muscle while stretching tape with 1/4 length removed; (**d**) Measure muscle length with tape; (**e**) divide the length of the tape into 4 equal parts; (**f**) attach to muscle while stretching tape with 1/4 length removed.

For the taping intervention, kinesio tape (Kinesiology 3NS Tape, TS, Gimpo-si, Korea) was used. Extensor digitorum longus taping method was attached from the origin (upper anterior 2/3 of the fibula) to the insertion (distal phalanges of the 2nd to 5th toes) of the extensor digitorum longus on the paretic side of the subject [14]. Tibialis anterior taping method was attached from the origin (2/3 of the outer upper part of tibia) to the insert (cuneiform bones) [17]. Once the length of muscles was measured with the subject in the supine position, the tape was cut, and then 1/4 of the tape was removed [24]. The proximal end of the tape was attached to the origin by dorsiflexion of the subject’s ankle joint at 90°, and the distal end of the tape was attached to the insertion. The tape was then applied to the skin by sweeping from the distal tape to the proximal tape [25]. Similarly, two layers of Kinesio tape were attached to the paretic side of muscles (Figure 1).

### 2.5. Measurement

#### 2.5.1. Balance Measurement

A balance-measuring device (BioRescue, RM Ingenierie, Rodez, France) was used to measure subject balance variables. This equipment measures the center of pressure movement length (cm), movement area (mm^2^), and limited stability (mm^2^) from a standing posture on a footrest.

In this study, limited stability (LOS) was measured as the subject moved their weight as much as possible in eight directions in the forward, backward, right, left, and diagonal directions indicated by the monitor from a standing position on the footrest and then the subject returned to the starting position. The right and left directions were classified as paretic and nonparetic sides. Previous studies show that LOS increases as the measured movement area in each direction increases [26]. In this study, measurements were made so that the foot did not deviate from the footrest during the measurement, and if it did move, a new measurement was made. A study assistant was always on standby to prevent falls when measuring LOS. The reliability of the measuring device was higher than 0.60 [27].

#### 2.5.2. Gait Measurement

Gait measuring equipment (G-walk, BTS Bioengineering, Milan, Italy) was used to measure gait variables. This device measures gait variables by placing a portable sensor in a belt pocket in the 5th lumbar region. The subjects walked at a comfortable pace along a 10 m walking path according to the evaluator’s instructions. The spatiotemporal gait variables of cadence, gait speed, stride length were measured [28,29]. A study assistant was always on standby to prevent falls when measuring gait variables. This gait measurement equipment can identify stroke patients and normal people [29]. Reliability has been demonstrated in the measurement of spatiotemporal gait variables in stroke patients [30]. The validity for cadence, speed, and stride length was 0.88–0.97, indicating excellent simultaneous validity [31].

### 2.6. Statistical Analyses

Statistical analyses were performed using the SPSS software (version 21.0; IBM, Armonk, NY, USA). The significance level was set at alpha ≤ 0.05 two tailed. The sample size was measured using the G-Power software (G-Power 3.1, Heinrich Heine-Universität, Düsseldorf, Germany).

General characteristics of the subjects in both groups were described by proportions and mean and standard deviations with differences between the groups compared using Chi Squared or independent two-sample *t*-tests. After pre-measurement, the normal distribution was assessed using the Shapiro–Wilk test. A two-way repeated-measures ANOVA test was used to evaluate the interventions. In the two-way repeated-measures ANOVA test, the paired t-test was used to determine significant differences over time, and the independent *t*-test was used to determine significant differences according to interactions.

## 3. Results

### 3.1. General Characteristics of the Subjects

The general characteristics of the study subjects are shown in Table 1. There were no statistically significant differences between the two groups based on descriptive variables (*p* > 0.05).

### 3.2. Results of Two-Way Repeated Measures of Limited Stability and Gait Variables

The results of the two-way repeated measures tests of the subjects’ limited stability and gait variables are shown in Table 2.

**Figure 2:**
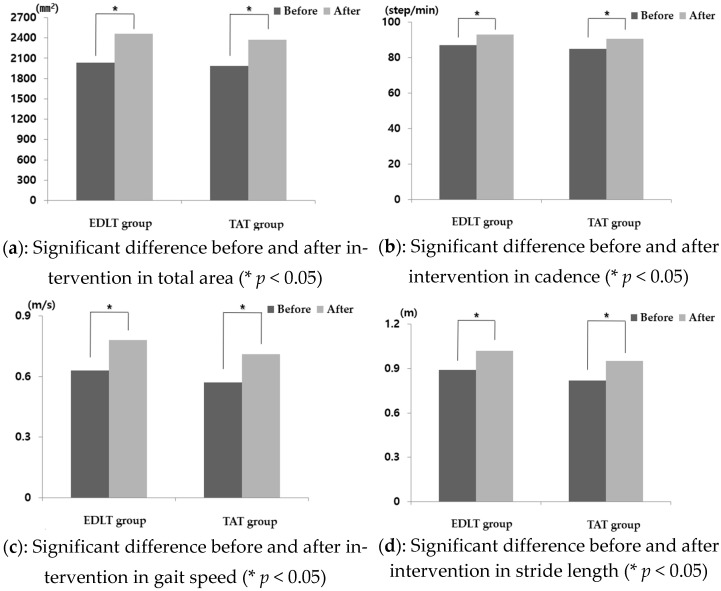
Limited of stability and variables changes are as follows: (**a**) change of total area (mm^2^); (**b**) change of cadence (step/min); (**c**) change of gait speed (m/s); (**d**) change of stride length (m).

Results of the within-subject tests showed that the limited stability were significantly increased in both groups after the intervention (*p* < 0.05), but there was no significant difference between the two groups (Figure 2a).

The results of the within-subject tests effects, cadence, gait speed, and stride length demonstrated significant increases in both groups after the intervention (*p* < 0.05), but there was no significant difference between the two groups (Figure 2b–d).

## 4. Discussion

This study aimed to compare the effects of EDLT and TAT on balance ability and gait performance in patients post-stroke. As a result of the study, gait speed was improved after taping was applied, but there was no difference in balance and gait performance between EDLT and TAT.

The increase in gait speed was not significantly between the EDLT and the TAT groups. Therefore, the results confirm and extend the results of previous studies reported on gait speed in stroke patients. Previous studies have shown that increasing dorsiflexion strength is required to improve gait speed [5].

In a previous study, dorsiflexion and eversion taping significantly improved gait speed, step length, stride length, cadence, and static and dynamic balance when compared with placebo taping and no taping [20,22,32]. Dorsiflexion and eversion taping are taping methods that directly apply mechanical correction to the ankle rather than assisting with muscle movement [20,22]. These types of methods prevent the talipes varus deformation caused by plantar flexion and improve balance and gait in patients post-stroke through proprioceptive input [22,32]. 

We observed the talipes varus deformation during the swing phase of gait, which may be attributed to the weakened extensor digitorum longus muscle, which minimized the ability to evert the ankle to counter inversion [9]. Kinesio taping can increase recruitment of the muscle’s motor units. This is more effective after 24 h than 72 h after taping attachment [33]. An increase in EMG of the tibialis anterior muscles correlates with an increase in gait speed [34]. We concluded that eversion (EDLT) or inversion (TAT) through taping did not affect the outcome. Pathological gait post-stroke is characterized by absent ankle joint dorsiflexion in the swing phase [35]. In this study, EDLT and TAT were attached to improve the dorsiflexion of the ankle joint to confirm the increase in gait speed. This study focused on dorsiflexion of the ankle joint and compared the effects of dorsiflexion with inversion and dorsiflexion with eversion. The stroke patients recruited for this study were those with ankle MAS grade of 1 or 2 and had spasticity in the ankle plantar flexor. Excessive TA activity may affect foot varus, but ankle plantar flexor was observed to be the main cause [9]. The difference in gait speed between TAT and EDLT may be affected by ankle spasticity. Therefore, it seems that a comparison between dorsiflexor taping and plantar flexor taping is necessary rather than TAT and EDLT in comparison between taping methods. On the other hand, weakness in dorsiflexion is the cause of a decrease in walking speed [36]. Two taping methods that promote the movement of dorsiflexion are possible ways to improve the walking performance of stroke patients.

We found no difference in balance performance between the two groups; however, balance significantly improved after taping. The taping method to improve dorsiflexion increases the range of motion of the dorsiflexion joint, so static balance can be improved [18,37]. Facilitation of muscle activity through taping reduces the center of pressure movement and maintains a standing position [38]. The TAT and EDLT performed in this study were taping methods performed for the purpose of increasing the range of motion of the dorsiflexion joint, and it is thought to be the result of improved posture control by contracting the corresponding muscles.

The lack of a difference in balance performance between the two groups may be explained by the application of various strategies instead of limiting treatment to the ankle joint to help maintain balance. An ankle strategy for posture control in a standing position is one of several exercise strategies. In patients post-stroke, the anterior–posterior ankle strategy is typically changed to a compensatory strategy owing to dorsiflexor weakness [39]. In patients post-stroke, the weight movement ability of the paretic leg was decreased compared to that of the non-paretic leg [40]. Notably, weight transfer ability uses a hip strategy rather than an ankle strategy [41]. In addition, patients post-stroke show visual dependence to maintain balance strategies due to motor and sensory impairments [42]. The EDLT and TAT methods used in this study were both ankle strategies. However, the two taping methods are not considered significantly different because a wide variety of balance strategies utilized in the clinical field are involved.

In a previous study, the ankle joint dorsiflexion tape was attached to the peroneus longus, peroneus tertius, extensor digitorum longus, and tibialis anterior [14]. Shortening of the plantar flexor muscle contributed to the appearance of talipes varus in patients post-stroke [9,13]. Although the peroneus longus is an evertor muscle, it could not be considered in this study because it is a plantar flexor muscle. Moreover, the peroneus tertius is not found in all people [43,44] and is considered a part of the extensor digitorum longus [44,45]. Therefore, this study focused on the extensor digitorum longus and tibialis anterior muscles, and the results are noteworthy because we compared the effects of individually taping each muscle.

Despite these results, this study had several limitations. The study confirmed the effects of taping ankle joint muscles for 24 h on balance and gait variables in patients post-stroke, but did not confirm the long-term effects. In a study on stroke patients, it was confirmed that the balance ability was improved 24 h after the ankle taping was applied [33]. This supports the results for this study on taping adhesion time. Although there is not enough evidence that the onset of the taping effect is after 24 h, taping showed a greater increase in handgrip strength at 24 h and 48 h than immediately after application [46]. However, since taping attachment for more than 48 h may cause skin problems, and the taping effect for 24 h may be insufficient to show the effect by supporting the function, it is still necessary to discuss the timing of the taping effect [47]. In addition, the effects of the applied taping methods on the activity of each muscle could not be specifically confirmed. In future studies, it will be necessary to directly test the activity of the muscle attached to the tape, and confirm whether the taping method has a positive effect on gait and balance variables over the long term in patients post-stroke. Although this study was a randomized controlled trial (RCT) design, it was difficult to compare the effects between the two groups due to the absence of a control group. Additional studies should be conducted considering control and sham groups to confirm clear effects.

## 5. Conclusions

The purpose of this study was to examine the effects of taping the extensor digitorum longus and tibialis anterior on balance and gait variables in patients post-stroke. Taping of the extensor digitorum longus or the tibialis anterior improved limited stability as well as gait variables in patients post-stroke. Short term taping to improve ankle dorsi flexion can improve balance and gait in patients post-chronic stroke. In the future, further research is needed on whether to add inversion or eversion functions for taping the ankle joint in stroke patients.

## Figures and Tables

**Table 1:** General characteristics.

Classification	EDLT Group (*n* = 20)	TAT Group (*n* = 20)	*p*
Gender (male/female)	13/7	13/7	1.000 ^a^
Paretic side (right/left)	12/8	11/9	1.000 ^a^
Type (infarction/hemorrhages)	13/7	15/5	0.731 ^a^
Disease duration (months)	14.10 ± 3.80	13.85 ± 3.69	0.834 ^b^
Age (years)	63.00 ± 7.62	63.45 ± 7.96	0.856 ^b^
Height (cm)	165.60 ± 8.59	162.60 ± 7.04	0.234 ^b^
Weight (kg)	70.20 ± 7.74	68.20 ± 6.29	0.376 ^b^
K-MMSE (score)	26.70 ± 1.75	27.05 ± 1.73	0.529 ^b^

All values are showed mean ± SD; ^a^ Chi-square test between two groups; ^b^ independent *t*-test between two groups; K-MMSE: Korean-mini mental state examination; EDLT group: Extensor digitorum longus taping; TAT group: Tibialis anterior taping.

**Table 2:** Two-way repeated measure analysis results of limited of stability and gait variables.

Classification	Before Intervention	After Intervention	Tests of Within-Subjects Effects		Tests of between-Subjects Effects	
			F	*p*	F	*p*
**Total area (mm^2^)**						
**EDLT ** **group**	2033.90 ± 255.81	2460.55 ± 378.12 ^a^	81.534	0.001 **	0.578	0.452
**TAT ** **group**	1987.55 ± 259.27	2370.75 ± 354.28 ^a^				
**Cadence (step/min)**						
**EDLT ** **group**	86.96 ± 11.73	92.92 ± 10.0 ^a^	58.515	0.001 **	0.266	0.609
**TAT ** **group**	84.81 ± 11.71	90.45 ± 12.02 ^a^				
**Gait Speed (m/s)**						
**EDLT ** **group**	0.63 ± 0.12	0.78 ± 0.08 ^a^	74.251	0.001 **	3.918	0.055
**TAT ** **group**	0.57 ± 0.12	0.71 ± 0.14 ^a^				
**Stride Length (m)**						
**EDLT ** **group**	0.89 ± 0.19	1.02 ± 0.11 ^a^	47.559	0.001 **	0.001	0.978
**TAT ** **group**	0.82 ± 0.21	0.95 ± 0.19 ^a^				

All values are showed mean ± SD, ** *p* < 0.01; ^a^ significantly different after the intervention (*p* < 0.05); EDLT group: Extensor digitorum longus taping; TAT group: Tibialis anterior taping.

## Data Availability

The corresponding author will provide data upon reasonable request.
